# High glucose and low specific cell growth but not mild hypothermia improve specific r-protein productivity in chemostat culture of CHO cells

**DOI:** 10.1371/journal.pone.0202098

**Published:** 2018-08-16

**Authors:** Mauricio Vergara, Mauro Torres, Andrea Müller, Verónica Avello, Cristian Acevedo, Julio Berrios, Juan G. Reyes, Norma A. Valdez-Cruz, Claudia Altamirano

**Affiliations:** 1 School of Biochemical Engineering, Pontificia Universidad Católica de Valparaíso, Valparaíso, Chile; 2 Institute of Chemistry, Pontificia Universidad Católica de Valparaíso, Valparaiso, Chile; 3 Center of Biotechnology, Universidad Técnica Federico Santa María, Valparaíso, Chile; 4 Institute of Physics, Universidad Técnica Federico Santa María, Valparaíso, Chile; 5 Departamento de Biología Molecular y Biotecnología, Instituto de Investigaciones Biomédicas, Universidad Nacional Autónoma de México, Ciudad de México, México; 6 Regional Center for Healthy Food Studies (CREAS) R17A10001, CONICYT REGIONAL, GORE Valparaiso, Chile; University College Dublin, IRELAND

## Abstract

In the biopharmaceutical sector, Chinese hamster ovary (CHO) cells have become the host of choice to produce recombinant proteins (r-proteins) due to their capacity for correct protein folding, assembly, and posttranslational modification. However, the production of therapeutic r-proteins in CHO cells is expensive and presents insufficient production yields for certain proteins. Effective culture strategies to increase productivity (q_p_) include a high glucose concentration in the medium and mild hypothermia (28–34 °C), but these changes lead to a reduced specific growth rate. To study the individual and combined impacts of glucose concentration, specific growth rate and mild hypothermia on culture performance and cell metabolism, we analyzed chemostat cultures of recombinant human tissue plasminogen activator (*rh*-tPA)-producing CHO cell lines fed with three glucose concentrations in feeding media (20, 30 and 40 mM), at two dilution rates (0.01 and 0.018 1/h) and two temperatures (33 and 37 °C). The results indicated significant changes in cell growth, cell cycle distribution, metabolism, and *rh*-tPA productivity in response to the varying environmental culture conditions. High glucose feed led to constrained cell growth, increased specific *rh*-tPA productivity and a higher number of cells in the G2/M phase. Low specific growth rate and temperature (33 °C) reduced glucose consumption and lactate production rates. Our findings indicated that a reduced specific growth rate coupled with high feed glucose significantly improves r-protein productivity in CHO cells. We also observed that low temperature significantly reduced q_p_, but not cell growth when dilution rate was manipulated, regardless of the glucose concentration or dilution rate. In contrast, we determined that feed glucose concentration and consumption rate were the dominant aspects of the growth and productivity in CHO cells by using multivariate analysis.

## Introduction

*In vitro* animal cell cultivation is currently the fastest growing area of the global pharmaceutical sector, particularly in the market of recombinant therapeutic proteins (r-proteins), with sales of more than $100 billion in 2013 [1]. In this highly sophisticated market, mammalian cells are key players for the commercial production of therapeutic proteins due to their potential for producing properly glycosylated and folded proteins [2,3]. Chinese hamster ovary (CHO) cells, which have proven to be robust and reliable on an industrial scale, are the workhorses of mammalian protein production [4]. However, lower production yields, when compared to other expression systems (e.g., bacteria), are one of the industry's main challenges in coping with increasing biopharmaceutical demand. This is why most efforts nowadays are focused on understanding the mechanisms involved in protein synthesis and the development of optimized processes to enhance productivity.

Many strategies aiming to enhance recombinant protein production focus on maximizing specific protein productivity while maintaining high viable cell density in culture for long periods. In this context, the operational conditions (e.g., temperature or medium composition) play a significant role in culture performance and proper handling of the cultures may indeed enable considerable increases in r-protein production [5–12].

Temperature is one of the most studied and important environmental variables in mammalian cell cultures. When reducing culture temperature from 37 °C to mild hypothermic (30–34 °C) conditions, cells significantly increase specific r-protein productivity (q_p_) in the majority of cases [13–18]. Although the specific reason for enhanced q_p_ remains uncertain, hypothermic culture conditions lead to changes in cellular machinery, which apparently favors enhanced r-protein production in batch mammalian cell cultures. Mild hypothermia of culture has been proved to cause cell cycle arrest in G0/G1 [19,20], improvements in the transcription and stability of foreign genes [17,21], and improvements in translation, folding and processing of proteins [22,23]. Moreover, mild hypothermia leads to a slowdown in growth and metabolism that is reflected in the decreased consumption of glucose and glutamine [24,25], lower production of lactate and ammonium [16,26], and a decreased specific growth rate [7,27].

Another key environmental variable impacting culture performance is media composition, particularly the nature and concentration of carbon and energy sources. Glucose is the main source of carbon and energy for the growth and maintenance of mammalian cells. From glucose metabolism, mammalian cells obtain essential intermediates, such as amino, fatty and nucleic acids, which serve as building blocks for synthesizing cellular components [28–30]. This is why a varying glucose concentration in media has multiple effects on the culture performance of mammalian cells, affecting specific growth rate, nutrient consumption rates, productivity and quality of r-proteins [30,31]. Nowadays, most industrially relevant culture media for mammalian cells contain a glucose concentration from 25 to 35 mM [29,32]. Therefore, 30 mM is the average glucose concentration for standard mammalian cell culture media, and concentrations below 20 mM are considered low [12,28], while concentrations above 40 mM are considered high [11,32], as compared with typical culture media. In cultures under very glucose-limited conditions (below 2 mM), cells have a drastically reduced intracellular concentration of ATP, amino acids and TCA cycle metabolites [31,33]. This leads to a lower q_p_ and deficient glycosylation of r-proteins in CHO cells [34,35]. However, despite the changes in cell metabolism, CHO cells cultured at low glucose concentration reduce lactate production and do not present detrimental changes at the transcriptome level [12]. In cultures under high glucose conditions (over 40 mM), cells present increased cAMP levels which activates relevant signaling pathways of carbon metabolism [10]. This results in enhanced r-protein production, but also results in reduced specific growth rate and changes in glycosylation, which might be undesirable [11,32,36,37]. Using high glucose media in mammalian cell cultures certainly has a positive effect on r-protein productivity. However, considerably high levels of glucose may be detrimental to cell growth and protein synthesis [11], causing cellular responses such as increased lactate production [38], generation of reactive oxygen species [39], endoplasmic reticulum stress [40] or apoptosis [41].

Manipulating both culture temperature and glucose concentration in media have proven to be successful strategies for improving specific productivity of r- proteins. However, changes in the culture environment, particularly in both operational variables, inevitably affect the specific growth rate. Most studies that have investigated the effect of changes in culture environment variables in CHO cells have been performed in batch cultures. However, using this culture modality, it is impossible to separate the effect of each variable from the resulting reduced specific growth rate and q_p_ [42]. To address this issue, chemostat culture has become a strategy to better understand how culture variables affect cellular behavior [43]. This is because it enables control of the specific growth rate by defining the dilution rate (D = F/V; F, feed flow; V, reaction volume) when reaching steady state (i.e., D = specific growth rate), maintaining the cells in a defined physiological state, and thus providing biologically reliable and homogeneous data of the cell population [34,42,44–46].

Previous studies in chemostat cultures have led to the identification of the impact of dilution rate [33] and glucose-limited media [35] on culture performance and product quality. Hayter et al. showed that, in glucose-limited chemostat cultures, q_p_ increased as specific growth rate increased, but nutrient limitation in cultures did not allow identification of a growth- r-protein production correlation [33,35]. Interestingly, the observed direct relationship between q_p_ and specific growth rate is contrary to most recent studies in other r-proteins. Berrios et al. reported an inverse relationship between the specific growth rate and specific productivity of *rh*-tPA, thereby suggesting that enhanced protein production was more related to changes in the specific growth rate than temperature or medium composition [34,47]. Subsequently, we investigated the effect of variations in culture temperature and specific growth rate on *rh*-tPA production. We demonstrated that lower dilution rates (lower specific growth rates) under mild hypothermic conditions promote higher specific productivity of *rh*-tPA, indicating that improved r-protein production is mainly modulated by changes in specific growth rate [42]. In view of these observations, it is still unclear which environmental variable is the actual modulator of productivity in CHO cell cultures. Therefore, it is relevant to investigate the metabolic responses against different levels of glucose and their effect on productivity under different metabolic states and culture temperature conditions to develop optimized culture strategies.

This study aims to elucidate the individual and combined effects of these three key operational variables on the modulation of r-protein production, cell growth, cell cycle distribution, and metabolism in an *rh*-tPA producing CHO cell line. To do so, we relied on the ability of chemostat culture set the specific growth rate to a desired value and manipulated the dilution rate to study the individual effect of culture temperature and glucose concentration in the feed. Based on our previous chemostat culture studies with a *rh*-tPA-producing CHO cell line [34,42,48], we evaluated the response of cell growth, cell cycle distribution, r-protein production and carbon metabolism at different temperatures (37 and 33 °C), glucose concentrations (20, 30 and 40 mM) and specific growth rates (0.018 and 0.010 1/h). Considering the complexity of the evaluated conditions, we incorporated a multivariate statistical analysis (MANOVA, PCA, and HCPC) to unravel the relative contribution of culture environment variables and compare the large data set generated from the chemostat cultures.

## Material and methods

### Cell line and culture medium

The *rh*-tPA-producing cell line (CHO TF 70R) was obtained from Pharmacia & Upjohn S.A. (Sweden) (a kind gift of Torsten Björlig). Glucose and glutamine-free SFM4CHO cell culture medium (HyClone, Logan, USA) supplemented with glucose (G7021, Sigma, USA) at different concentrations and 6 mM glutamate (G8415, Sigma, USA) was used as growth medium for all cultures.

### Chemostat cultures

Before the chemostat culture experiments, cells were sub-cultured every 48 h in batch systems using T-75 flasks and scaled up in spinner flasks (Techne, UK) by reseeding at 0.25 × 10^6^ cells/mL in fresh growth medium. To evaluate the effect of mild hypothermia, cells were acclimatized to low temperature by successive reduction of the culture temperature. Cells were sub-cultured every 48 h at 35 °C and then at 33 °C for 3 culture passages in each temperature [49]. The gradual reduction of temperature enabled us to maintain high cell viability during the process.

Chemostat culture experiments were performed in spinner flasks (Techne, UK) specially conditioned for continuous operation with a working volume of 150 mL. Cultures were initially operated in batch mode for 48 h and were then supplied with sterile feed throughout the period of operation [34]. All cultures were incubated in a Forma Scientific CO_2_ Incubator (Thermo Fisher Scientific Inc., USA) at 50 rpm, 5% CO_2_, and 95% relative humidity.

To evaluate different conditions of temperature (High Temperature: 37 °C or Low Temperature: 33 °C), dilution rate (Low-D: 0.010 1/h or High-D: 0.018 1/h), and glucose concentration (Low: 20 mM, Intermediate: 30 mM and High: 40 mM), twelve experiments were performed in duplicate. Cultures were sampled every 24 h for viable cell quantification and further analytical measurements. Cultures were considered to reach steady state (SS) when, after at least four residence times, both the number of viable cells and lactate concentration were constant in two consecutive samples.

### Analytical measurements

Cell quantification and viability were determined by the trypan blue exclusion method (T8154, Sigma, USA) (1:1 mixture of 0.2% trypan blue in saline and cell sample) using a hemocytometer (Neubauer, Germany). Glucose and lactate were measured with an automatic biochemistry analyzer (YSI 2700, Yellow Springs Inc., USA). Extracellular *rh*-tPA was quantified by enzyme immunoassay (Trinilize tPA-antigen kit, Tcoag Ltd., Ireland).

### Cell cycle analysis

For cell cycle analysis, 1.0 × 10^6^ cells were fixed by mixing re-suspended cells in 200 μL PBS with 1.8 mL cold 70% (v/v) ethanol. Samples were stored at -20 °C until measurement, according to the Stalk Institute protocols (“http://fccf.salk.edu/protocols/cellcycle.php”) and Krishan (1975) [50]. Cells were mixed with 1 mL propidium iodide solution, containing 40 μg/mL of propidium iodide (#P3566, Molecular Probes^™^, USA) and 3.8 mM sodium citrate (S1804, Sigma, USA). After incubation for 3 h at 4 °C with 50 μL of stock RNase A solution, the cell cycle was analyzed using a Beckman Coulter FC500 flow cytometer (Beckman Coulter Life Sciences, USA) and Cylchred software from Cardiff University [51]. Results are presented as the percentage of cells in phases G1/G0, S, or M of the cell cycle.

### Estimation of specific rates at steady state

Specific growth rate (μ) was calculated from the mass balance within the bioreactor:

$$\mu =D(N_{t}/N_{v})$$ (1)

where N_*v*_ is the concentration of viable cells (10^6^/mL), N_*t*_ is the concentration of total cells (10^6^/mL), and D is the dilution rate of the culture (1/h) [42,48].

Specific rates of production or consumption (Eq 2) of metabolite i (q_i_) were calculated from the mass balance within the reactor:

$$q_{i}=D(\left(C_{i}^{i}-C_{i}^{0}\right)/N_{v})\times 10^{9}$$ (2)

where $C_{i}^{i}$ is the concentration of i in the inlet (mmol/L), $C_{i}^{0}$ is the concentration of i in the outlet (mmol/L), N_*v*_ is the concentration of viable cells (10^6^/mL), and D is the dilution rate of the culture (1/h).

### Statistical analysis

All culture experiments were performed in duplicate, and two independent samples were taken at each time point for every culture, with analytical measurements carried out separately. Values of kinetic and stoichiometric parameters are expressed as the mean ± standard error (SE). All statistical analyses were performed using R software (version 3.1.) [52]. Statistical significance of the variations caused by the different experimental conditions was evaluated by multivariate analysis of variance (MANOVA) (using culture temperature– 2 levels, dilution rate– 2 levels, and glucose concentration– 3 levels as factors) followed by multiple comparison tests (Tukey HSD test) with normally distributed data. Variance homogeneity and normal distribution of residuals were assessed with the Shapiro-Wilk test and visual inspection of the normal-quantile plot in order to validate the MANOVA’s assumptions. The threshold for statistical significance was p < 0.05. Multivariate principal components analysis (PCA) and hierarchical clustering analysis (HCPC) were performed for the main physiological parameters (i.e., 5 variables) and the cell cycle analysis results using the FactoMineR package [53].

## Results

**Fig 1:**
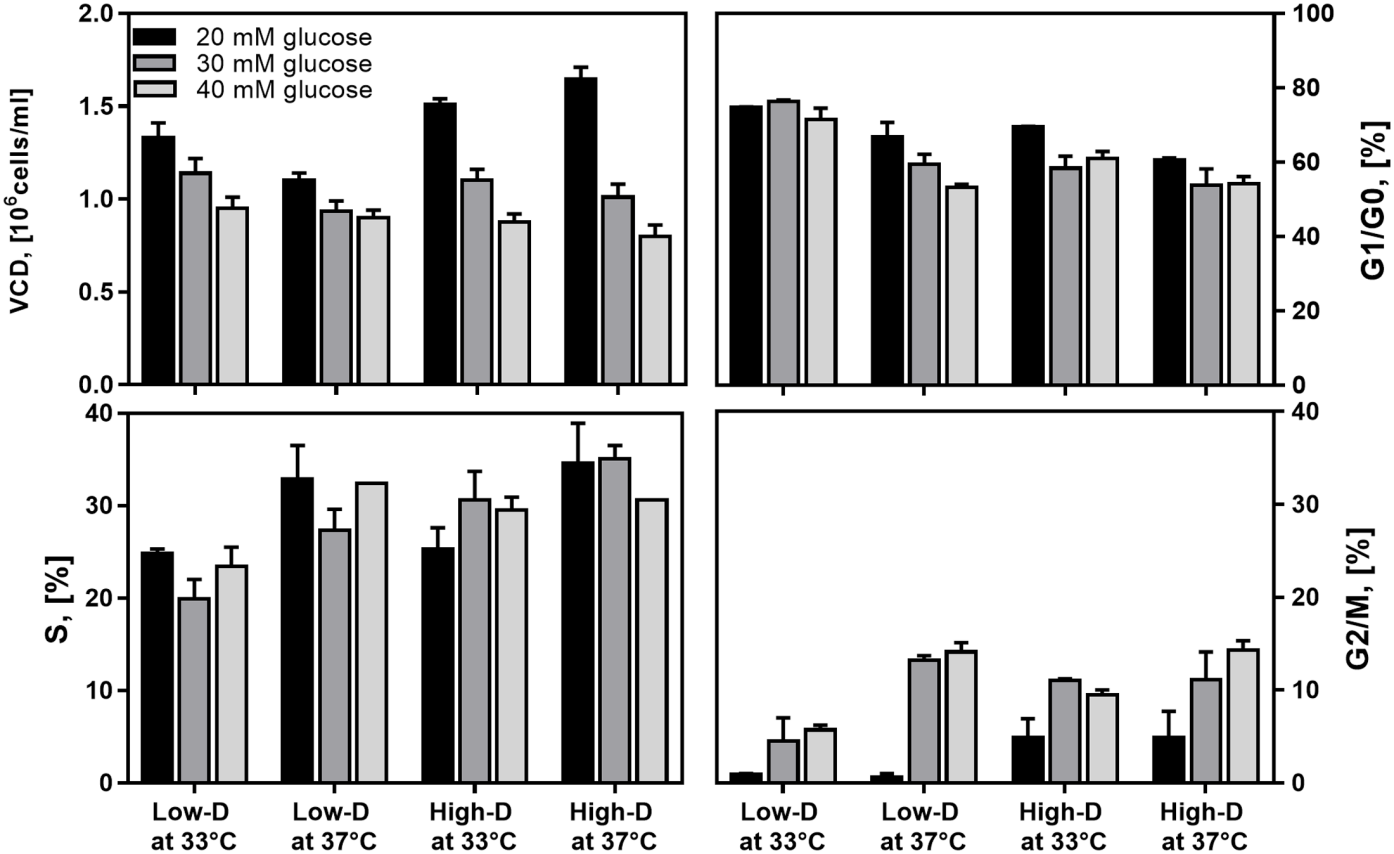
Impact of dilution rate, culture temperature and feed glucose concentration on viable cell concentration and cycle distribution in CHO cell chemostat cultures at steady state. (*Black*) 20 mM glucose; (*Dark grey*) 30 mM glucose; (*Light grey*) 40 mM glucose.

**Fig 2:**
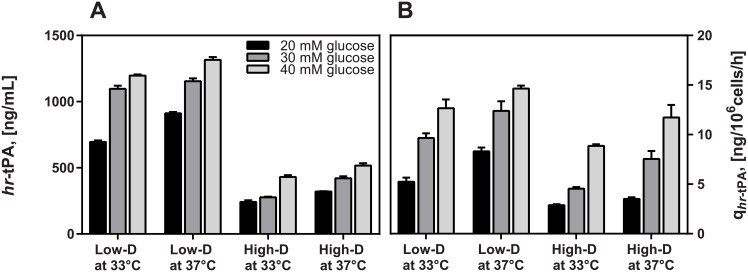
Impact of dilution rate, culture temperature and feed glucose concentration on *hr*-tPA production and specific *hr*-tPA productivity in CHO cell chemostat cultures at steady state. (*Black*) 20 mM glucose; (*Dark grey*) 30 mM glucose; (*Light grey*) 40 mM glucose.

**Fig 3:**
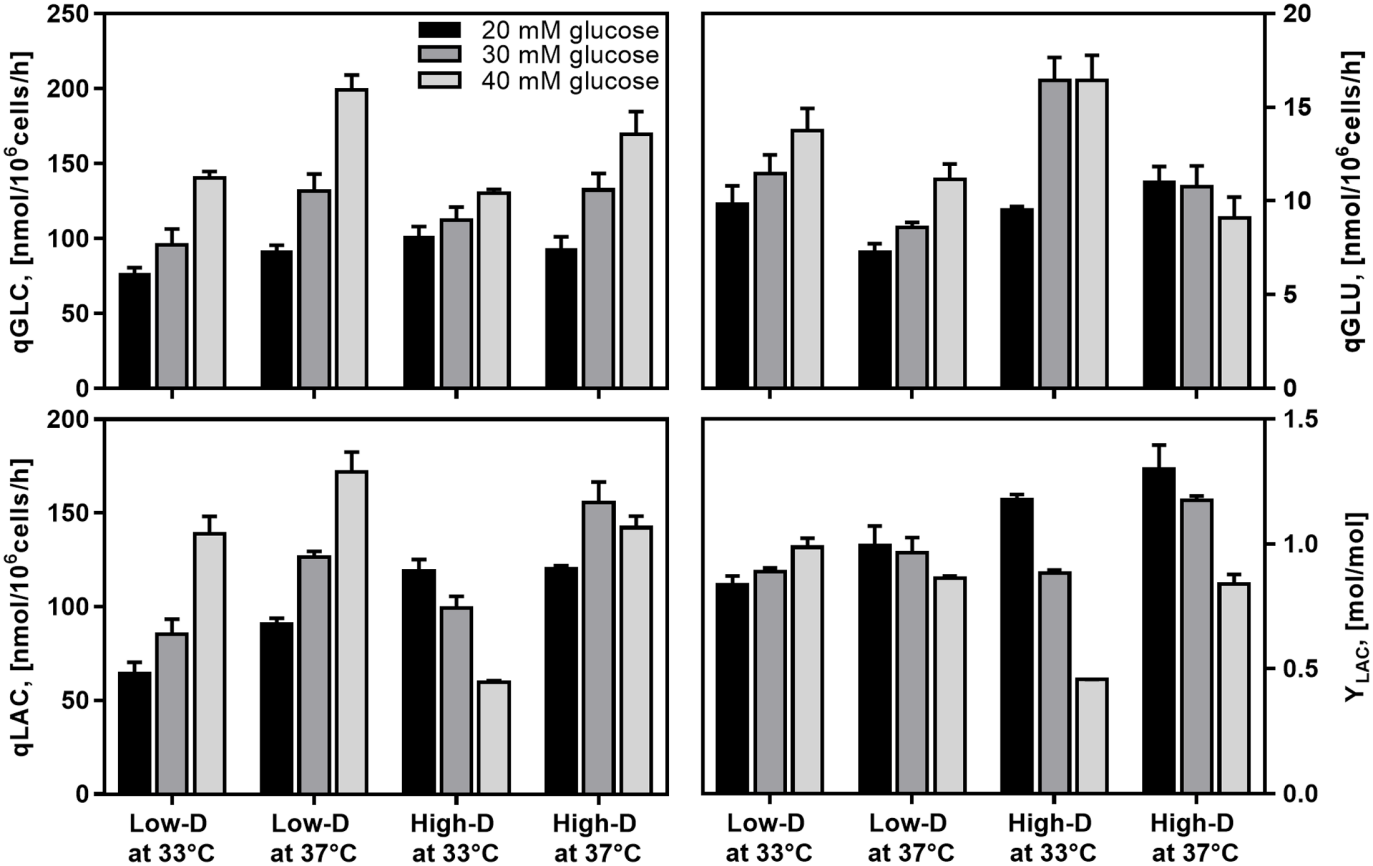
Impact of dilution rate, culture temperature and feed glucose concentration on glucose, glutamate and lactate metabolism in CHO cell chemostat cultures at steady state. (*Black*) 20 mM glucose; (*Dark grey*) 30 mM glucose; (*Light grey*) 40 mM glucose.

In this study, we investigated the differential effects of dilution rate, culture temperature, and medium glucose concentration on culture performance and cell metabolism of the *rh*-tPA-producing CHO cell line. A series of chemostat cultivations at two dilution rate levels (0.01 and 0.018 1/h as Low-D and High-D, respectively), two culture temperature (33 and 37 °C) and three different feed stream glucose concentrations (20, 30 and 40 mM) were performed in duplicate. All chemostats at each experimental condition reached steady state after four residence times and cell viability remained above 90% for all cultures (Table 1). From each cultivation, cell growth, cell cycle distribution, metabolism, and *rh*-tPA productivity were evaluated by calculating the corresponding physiological parameters (Figs 1, 2 and 3). Given the nature of the data in this study, mainly due to the multiplicity of evaluated conditions, the results were analyzed using multivariate statistical analysis techniques (MANOVA, PCA, and HCPC) and compared using a multiple comparison post hoc test.

**Table 1:** Viable cell concentration and viability in CHO cell chemostat cultures at steady state.

	Viable cell concentration, [10^6^ cell/mL]				Specific cell growth rate, [1/h]				Cell viability, [%]			
	Low-D		High-D		Low-D		High-D		Low-D		High-D	
	33°C	37°C	33°C	37°C	33°C	37°C	33°C	37°C	33°C	37°C	33°C	37°C
20 mM glucose	1.33 ± 0.11	1.10 ± 0.06	1.51 ± 0.04	1.65 ± 0.09	0.011 ± 0.0	0.011 ± 0.0	0.019 + 0.0	0.019 + 0.0	92.6 ± 0.9	92.9 ±0.8	94.7 ± 1.1	94.0 ± 1.1
30 mM glucose	1.14 ± 0.11	0.94 ± 0.08	1.10 ± 0.08	1.01 ± 0.10	0.011 ± 0.0	0.011 ± 0.0	0.019 + 0.0	0.019 + 0.0	92.0 ± 0.8	92.6 ±0.6	93.6 ± 0.8	94.8 ± 0.7
40 mM glucose	0.95 ± 0.08	0.90 ± 0.06	0.88 ± 0.06	0.80 ± 0.08	0.011 ± 0.0	0.011 ± 0.0	0.019 + 0.0	0.019 + 0.0	91.8 ± 0.7	93.2 ± 0.6	93.6 ± 0.6	94.0 ± 0.5

### Impact of culture conditions on cell growth and cycle distribution

Cell growth in the *rh*-tPA-producing CHO cell line was strongly affected by variations in culture conditions (Fig 1 and Table 1). The MANOVA reveals that cell growth levels were significantly different between the two dilution rates, the three feed glucose concentrations, and the two culture temperature conditions (Table 2). Maximum cell densities were found at low glucose concentration (20 mM) and High-D, both at 33 and 37 °C (Fig 1A). An increase in glucose concentration resulted in a significant decrease in cell density levels, regardless of dilution rate or culture temperature, as indicated by Tukey’s post hoc test. Increasing the glucose concentration from 20 to 40 mM resulted in the most significant decline in cell density, decreasing by 29% at Low-D and 33 °C (p < 0.05) and by 18% at 37 °C (p < 0.05), while decreasing by 42% at High-D and 33 °C (p < 0.05) and by 51% at 37 °C (p < 0.05). Additionally, Tukey’s post hoc analysis indicated that cell growth significantly decreased as glucose concentration was increased from 20 to 30 mM and from 30 to 40 mM. Therefore, an increase in glucose concentration adversely affected the cell density levels. On the other hand, a decrease in dilution rate had a dissimilar effect depending on the glucose concentration in feeding media. At 20 mM glucose, the cell concentration at Low-D decreased by 12% at 33 °C (Tukey’s post hoc test, p < 0.05) and by 33% at 37 °C (Tukey’s post hoc test, p < 0.05) as compared to High-D. At 30 mM glucose, there were no significant changes in cell concentration. Meanwhile, at 40 mM glucose and Low-D the cell concentration increased by 9% at 33 °C (Tukey’s post hoc test, p < 0.05) and by 13% at 37 °C (Tukey’s post hoc test, p < 0.05) as compared to High-D at 33 °C and 37 °C, respectively. Thus, the positive or negative effect of changes in dilution rate on cell growth is highly dependent on the glucose concentration in feeding media. Mild hypothermia slightly improved the viable cell concentration in most of the evaluated conditions, except for cultures at High-D and 20 mM glucose. However, these improvements were not statistically significant.

**Table 2:** Individual and combined impact of dilution rate, culture temperature and feed glucose concentration on the physiological parameters in chemostat cultures at steady state (MANOVA; n = 3).

	Dil		Temp		GLC		Dil:Temp		Dil:GLC		Temp:GLC		Dil:Temp:GLC	
	F-value	p-value	F-value	p-value	F-value	p-value	F-value	p-value	F-value	p-value	F-value	p-value	F-value	p-value
Xv	8.17	*	6.33	*	79.27	***	4.94	*	15.88	***	0.83	n.s	2.80	n.s
qGlc	0.02	n.s	27.64	***	62.74	***	3.60	n.s	4.15	*	6.68	*	0.04	n.s
qLac	0.65	n.s	105.36	***	19.29	***	2.81	n.s	56.87	***	11.79	**	7.66	**
Ylac	3.68	n.s	34.14	***	43.36	***	19.64	***	45.67	***	0.41	n.s	9.21	**
qGlu	11.45	**	35.35	***	12.25	**	1.10	n.s	2.97	n.s	6.18	*	5.79	*
qtPA	119.30	***	42.77	***	122.71	***	0.35	n.s	1.96	n.s	0.67	n.s	1.95	n.s
G1.G0	29.24	***	58.62	***	11.91	**	7.49	*	2.46	n.s	0.76	n.s	2.46	n.s
S	9.60	**	23.92	***	0.27	n.s	1.43	n.s	3.64	n.s	0.68	n.s	0.95	n.s
G2.M	9.41	**	16.03	**	31.56	***	4.86	*	0.55	n.s	4.77	*	2.02	n.s

Dil: dilution rate; Temp: culture temperature; GLC: glucose concentration in feeding.

***, **, * and n.s correspond to p < 0.001, p < 0.01, p < 0.05 and non-significant, respectively. F-values were obtained through Wilks'λ test and used to calculate the p-values. High F-values result in low p-values and indicate the statistical significance of parameter variation.

Cell cycle distribution was significantly different among the distinct culture conditions as revealed by MANOVA (Table 2). While the percentage of cells in G1/G0 and S phases was mainly affected by the variation in culture temperature and dilution rate, the percentage of cells in G2/M was predominantly affected by changes in glucose concentration (Fig 1). The largest percentage of cells in G1/G0 phase was found at Low-D and 33 °C for all glucose concentrations (Fig 1B). The percentage of cells in G1/G0 phase was significantly lower as culture temperature and/or dilution rate increased (Tukey’s post hoc test, p < 0.05). The percentage of cells in S phase followed the opposite trend of those in G1/G0, reaching a maximum level at High-D and 37 °C for all glucose concentrations (Fig 1C). The percentage of cells in S phase significantly decreased with culture temperature reduction (Tukey’s post hoc test, p < 0.05). Finally, the largest proportion of cells in G2/M was found at 40 mM glucose and 37 °C, both at Low-D and High-D. The percentage of cells in G2/M phase was significantly lower as glucose concentration decreased in culture (Tukey’s post hoc test, p < 0.05). The fraction of cells in G2/M was also reduced as temperature decreased, at 30 mM glucose (Low-D), and at 40 mM glucose. Therefore, mild hypothermia and Low-D increased the accumulation of cells in G1/G0 phase and reduced the number of cells in S and G2/M phases. However, higher glucose concentrations (30 and 40 mM) increased the accumulation of cells in G2/M phase and reduced the number of cells, mainly in G1/G0 at 37 °C, both at High-D and Low-D.

### Impact of culture conditions on r-protein production

Production of *rh*-tPA in CHO cell cultures was assessed and compared in all experimental conditions by calculating specific *rh*-tPA productivities (q_tPA_) (Fig 2). According to the MANOVA, both the *rh*-tPA production and specific productivity varied significantly between the two dilution rates, three glucose concentrations and two culture temperatures (Table 2). Maximum *rh*-tPA production and q_tPA_ were found at 40 mM glucose, Low-D and 37 °C (Fig 2A and 2B). *rh*-tPA production and productivity were significantly higher as glucose concentration and culture temperature increased and as dilution rate decreased (Tukey’s post hoc test, p < 0.05). In fact, an increase in glucose concentration from 20 to 40 mM represented an improvement of q_tPA_ by 41% at Low-D and 33 °C (p < 0.05) and by 57% at 37 °C (p < 0.05), while at High-D, an increase of 33% at 33 °C (p < 0.05) and 30% at 37 °C (p < 0.05) were observed. Therefore, production of *rh*-tPA in CHO cells was governed by glucose concentration and dilution rate, where a higher glucose concentration and lower dilution rate positively impacted *rh*-tPA productivity in cultures. On the other hand, q_tPA_ was significantly lower at 33 °C at the three glucose concentrations and two dilution rates. A lesser impact of mild hypothermia on *rh*-tPA productivity was observed at 40 mM of glucose (with 32% reduction at High-D) than at 30 mM and 20 mM (around 45% reduction). Thus, mild hypothermia reduced *rh*-tPA productivity, regardless of the glucose concentration and dilution rate in cultures. A lower impact of mild hypothermia on productivity was observed at 40 mM of glucose (30% of reduction), than at 30 mM and 20 mM (around 45% reduction).

### Impact of culture conditions on metabolic behavior

Glucose, lactate, and glutamate metabolism and changes in their consumption and production due to culture conditions were evaluated by the residual concentration of glucose, lactate, and glutamate in chemostat cultures at steady state (Table 3) and by calculating their corresponding specific consumption and production rates for all conditions (Fig 3). MANOVA indicated that specific glucose and glutamate consumption, and lactate production rates were significantly different between the two culture temperatures and the three glucose concentrations (Table 2).

**Table 3:** Residual concentration of glucose, glutamate and lactate in chemostat cultures at steady state.

	Glucose, [mM]				Glutamate, [mM]				Lactate, [mM]			
	Low-D		High-D		Low-D		High-D		Low-D		High-D	
	33°C	37°C	33°C	37°C	33°C	37°C	33°C	37°C	33°C	37°C	33°C	37°C
20 mM glucose	9.8 ± 0.1	9.9 ± 0.1	11.5 ± 0.6	11.5 ± 0.6	4.7 ± 0.1	5.2 ± 0.1	5.2 ± 0.1	5.0 ± 0.1	8.5 ± 0.4	10.0 ± 0.9	10.0 ± 0.4	11.0 ± ‘.4
30 mM glucose	19.2 ± 0.6	17.8 ± 0.5	23.2 ± 0.2	22.6 ± 0.1	4.7 ± 0.1	5.2 ± 0.1	5.0 ± 0.1	5.4 ± 0.1	9.6 ± 0.4	11.8 ± 0.6	6.0 ± 0.1	8.7 ± 0.1
40 mM glucose	26.6 ± 0.6	22.1 ± 0.2	33.7 ± 0.6	32.5 ± 0.1	4.7 ± 0.1	5.0 ± 0.1	5.2 ± 0.1	5.6 ± 0.1	13.1 ± 0.1	15.4 ± 0.4	2.9 ± 0.3	6.3 ± 0.3

Dil: dilution rate; T: culture temperature; GLC: glucose concentration in feed.

A maximum specific glucose consumption rate (q_Glc_) was found in cultures at 40 mM glucose, Low-D, and 37 °C (Fig 3). The q_Glc_ was significantly lower with the lower glucose concentrations, as expected. Tukey’s post hoc analysis showed that q_Glc_ was significantly lower as glucose concentration decreased from 40 to 30 mM and from 30 to 20 mM glucose. As the feed glucose concentration decreased from 40 to 20 mM, the q_Glc_ declined by 46% at Low-D and 33 °C (p < 0.05) and by 54% at 37 °C (p < 0.05), while decreasing by 22% at High-D and 33 °C (p < 0.05) and by 45% at 37 °C (p < 0.05). In contrast, the variation in dilution rate showed no impact on q_Glc_ for all glucose concentrations and culture temperatures. Meanwhile, mild hypothermia only had a significant impact on q_Glc_ in cultures at 40 mM glucose, decreasing by 30% at Low-D and 23% at High-D at 33 °C, when compared to 37 °C.

A maximum specific glutamate consumption rate (q_Glu_) was found in cultures at 30 mM glucose, High-D, and 33 °C (Fig 3). The increase in glucose concentration from 20 to 30 mM resulted in a significant increase in q_Glu_ in cultures at 33 °C, both at Low-D and High-D (Tukey’s post hoc analysis, p < 0.05). The q_Glu_ at 30 or 40 mM glucose showed almost the same levels in cultures at 33 °C. In turn, an increase in culture temperature decreased the q_Glu_ significantly in all culture conditions, except for cultures at High D and 20 mM glucose, which showed a higher q_Glu_. These results showed that cells consumed more glutamate under mild hypothermia and at high glucose concentrations. However, these conditions were far from resulting in higher cell growth or *rh*-tPA production.

Lactate production was noticeably influenced by changes in culture conditions. A maximum specific lactate production rate (q_Lac_) was found in cultures at 40 mM glucose, Low-D, and 37 °C (Fig 3), which was in line with the condition presenting the highest q_Glc_. Higher glucose concentration resulted in a significant increase in q_Lac_ at Low-D, while at High-D, it decreased at 33°C and increased at 37 °C. On the other hand, lower culture temperature significantly reduced q_Lac_ in all culture conditions, except for the cultures at 20 mM and High-D, as indicated Tukey’s post hoc analysis. The impact of dilution rate on the q_Lac_ was strongly dependent on the feeding media glucose concentration. While cultures at 20 mM glucose showed higher q_Lac_ at High-D, the opposite was observed in cultures at 40 mM, where higher q_Lac_ values were found at Low-D.

Additionally, the yield of lactate from glucose (Y_Lac/Glc_) was calculated to assess the metabolic state of cells (Fig 3D). An increase in glucose concentration significantly reduced Y_Lac/Glc_, but only for the cultures at High-D. The effect of dilution rate on Y_Lac/Glc_ was strongly dependent on the medium glucose concentration, while the low temperature had no significant impact on Y_Lac/Glc_. An interesting finding was that the most efficient carbon utilization condition was observed at high glucose concentration.

### Multivariate analysis of cell behavior under different culture conditions

**Fig 4:**
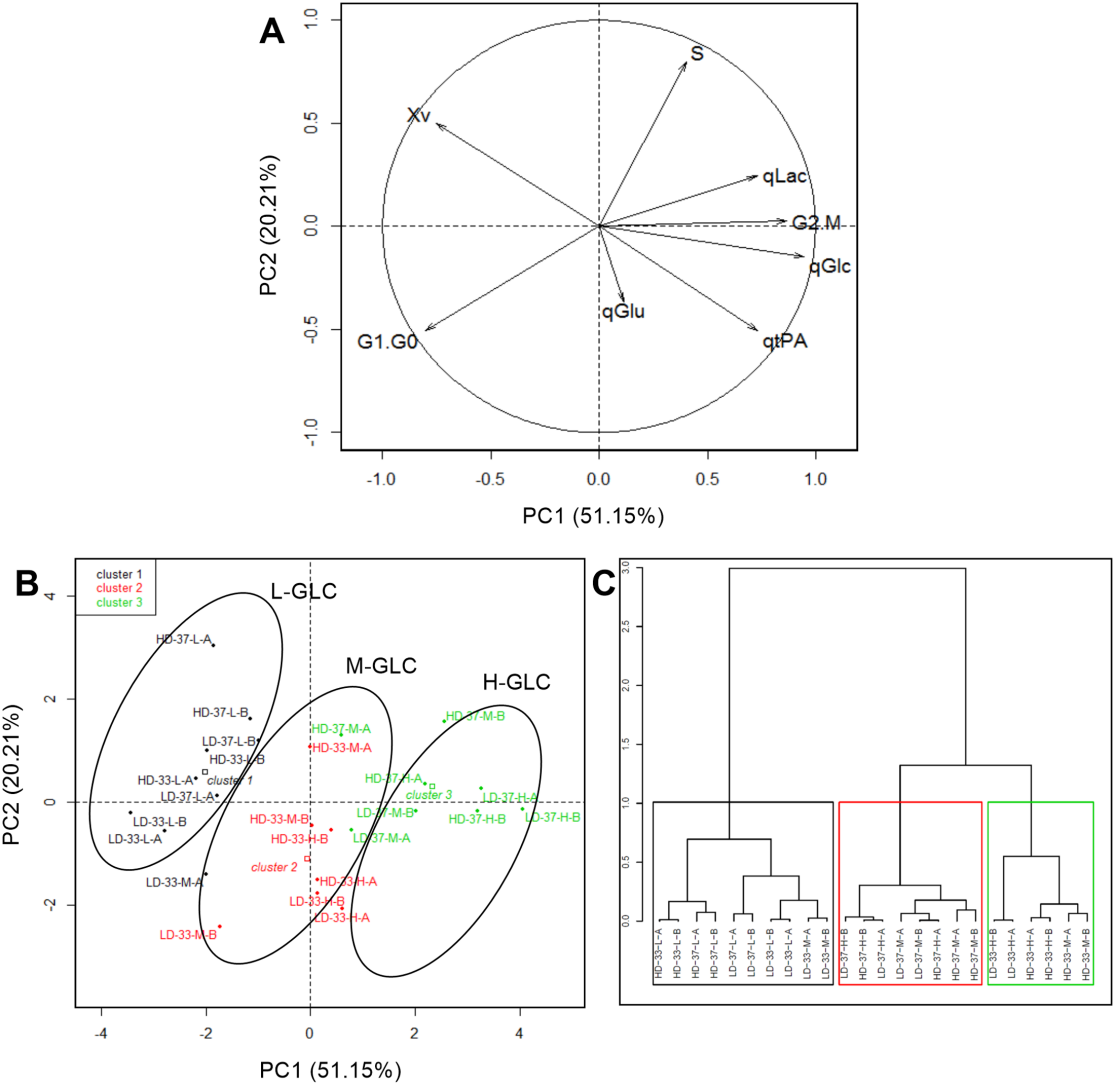
Principal component analysis (PCA) calculated using 10 physiological parameters of the 24 chemostat culture experiments. A corresponds to the ordination of parameters and correlation circle. B corresponds to the position of observations in the ordination where conditions of feed glucose concentration (i.e. H, M and L), dilution rate (i.e. HD and LD), temperature (i.e. 37 and 33) and each biological replicate (i.e. A and B) are represented, showing the effect of feed glucose concentration with circles. C corresponds to a hierarchical classification tree based on principal components and the different clusters evaluated using 1000 iterations. Xv: viable cell concentration; qGlu: specific glucose consumption; qLac: specific lactate production; Ylac: glucose on lactate yield; qGlu: specific glutamate consumption; qtPA: specific *rh*-tPA production; G1.G0, S and G2.M: percentage of cells placed in the corresponding point of cell cycle. PCA and hierarchical classification were performed using the “FactoMineR” package [53].

**Fig 5:**
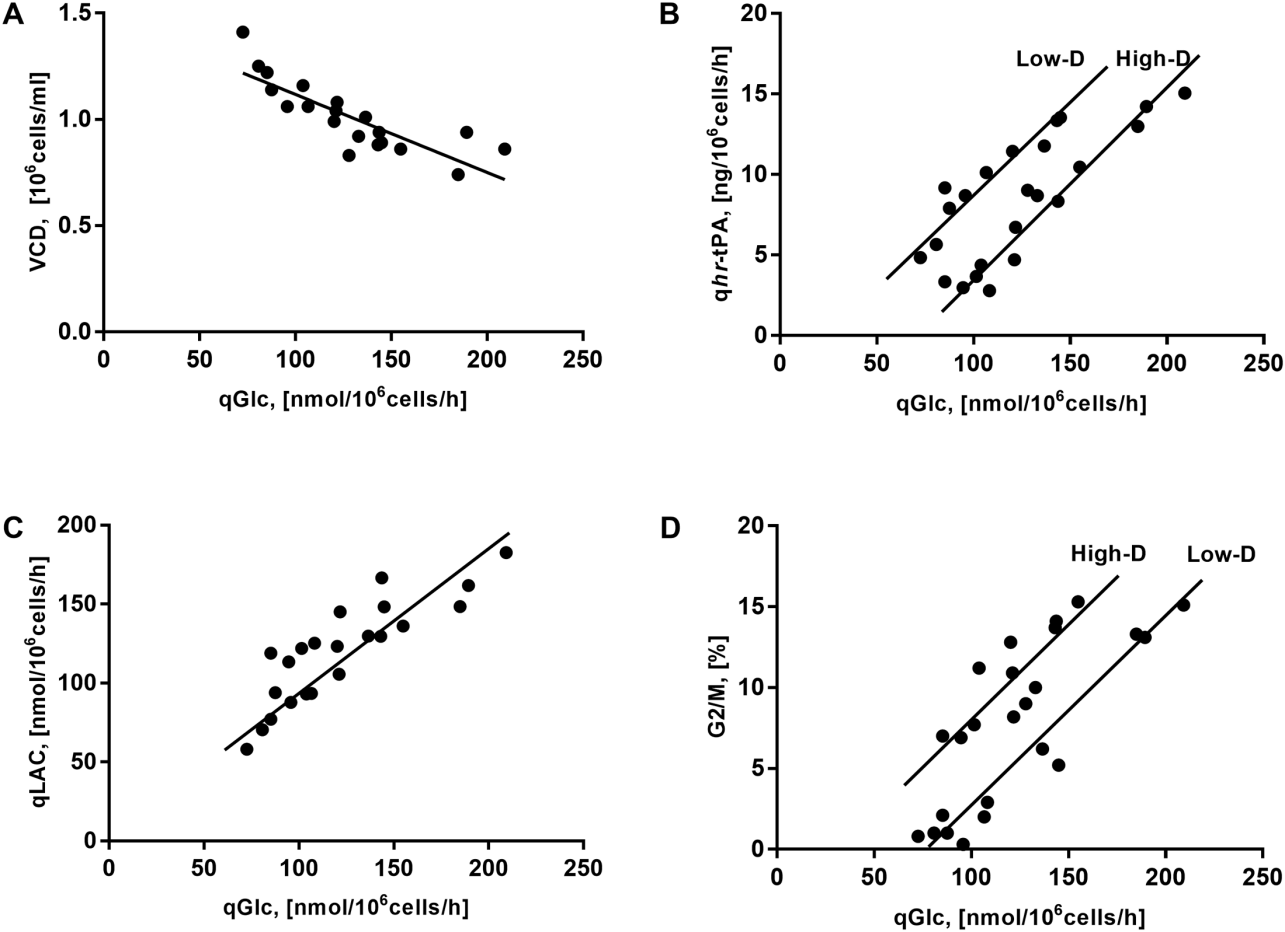
Correlation of physiological parameters and q_GLC_ observed by PCA in CHO cells cultures at different glucose concentrations, dilution rates and culture temperatures. A corresponds to the correlation between viable cell concentration and q_GLC_ (Pearson’s correlation R = -0.83, p < 0.05). B corresponds to the correlation between q_*rh*-tPA_ and q_GLC_ (Pearson’s correlation R = 0.94, p < 0.05 for High-D, and R = 0.91, p < 0.05 for Low-D). C corresponds to the correlation between q_LAC_ and q_GLC_ (Pearson’s correlation R = 0.74, p < 0.05). D corresponds to the correlation between G2/M and q_GLC_ (Pearson’s correlation R = 0.81, p < 0.05 for High-D, and R = 0.98, p < 0.05 for Low-D). The pearson correlation coefficients were calculated using the R software [52].

To elucidate the influence of physiological parameters on culture performance and cell behavior of the *rh*-tPA-producing CHO cell line, a principal component analysis (PCA) and a hierarchical clustering analysis (HCPC) were performed (Fig 4). Eight variables including viable cell density, q_GLC_, q_LAC_, q_GLU_, q_*hr*-tPA_, G1/G0, S, and G2/M of the 24 chemostat cultures were considered for these analyses. In the PCA, principal components 1 and 2 (PC1 and PC2, respectively) represented 71.4% of total variance, in which q_GLC_ was the variable with the highest contribution to the total variance. To provide insight on the relationship among the parameters evaluated, the corresponding data were plotted in a two-dimensional diagram considering PC1 and PC2 (Fig 4A). From this analysis, q_GLC_ was strongly and positively correlated with q_*hr*-tPA_, q_LAC_, and G2/M, while it was negatively associated with cell growth and, to a lesser extent, with G1/G0. These results were corroborated by calculating Pearson correlations, which showed higher correlations between q_GLC_ and the mentioned cell concentration, q_*hr*-tPA_, q_LAC_ and G2/M (Fig 5). Along with this, data analysis showed almost no relationship between q_*hr*-tPA_ and G1/G0. Although these results are different from those reported previously, in which an increased r-protein production might be associated with a cell cycle arrest in G1 [19,20], these studies did not consider the decrease of specific cell growth, nor the relationship between increased r-protein production and cell cycle arrest in the G2/M phase. In contrast, the HCPC reflected the impact of environmental culture conditions on the formation of clusters (Fig 4B). Glucose concentration was the condition with the largest impact on the main cluster division, while changes in dilution rate and culture temperature do not allow a clear separation of clusters. Therefore, for glucose, both its concentration in the feed and consumption by cells, were the aspects that controlled physiological parameters variability in the studied CHO cell cultures.

## Discussion

Temperature and media composition are two of the most studied environmental conditions in CHO cell cultures and changes in these conditions have a broad impact on cell growth, metabolism and r-protein production [5,54]. Nowadays, the effect of mild hypothermia and glucose concentration on recombinant CHO cells has been widely reported in batch cultures [11,19,32,55]. However, such a culture modality does not allow separation of the effect of the environmental variable from the effect of altered specific cell growth due to these changes. Thus, we present in this study the analysis of individual and combined effects of temperature, feed glucose concentration and dilution rate on culture performance and cell metabolism using chemostat cultures and multivariate statistical techniques.

By increasing glucose concentration in the feed media from 20 to 40 mM, cell growth was negatively affected. Glucose is certainly a key carbon source for cell growth, and changes in its concentration in the culture medium are proven to regulate cell growth [56–58]. A cell growth constraint has been observed in CHO cell batch cultures when glucose concentration increases beyond 25 mM [10,11,32]. In contrast, Hayter et al. [33] and Berrios et al. [34] showed an increase in viable cell density in the range of 2.5 to 10 mM in two different recombinant CHO cell lines in response to increased feed glucose concentration in chemostat cultures. In accordance with our previous results in chemostat cultures at 10 mM glucose [42], we observed a decrease in viable cell density as glucose concentration increased from 20 to 40 mM, regardless of dilution rate and culture temperature. The specific mechanism of cell growth regulation by changes in glucose concentrations still remains unclear. Lin et al. [10] provided evidences that high glucose media induces cell growth attenuation through two signaling pathways. High glucose in medium increases cAMP levels and activates two signaling pathways (cAMP/PKA and MAPK) involved in reducing the growth rate of CHO cells [10]. Furthermore, Lee et al. [32] reported an attenuation of several enzymes along the TCA cycle, explaining that, under high-glucose conditions, pyruvate is mainly converted to lactate, which is toxic for cells. In high glucose media, an upregulation of genes involved in oxidative phosphorylation and anti-oxidative pathways has also been observed in CHO cells [11] and other mammalian cell lines [59,60]. This response has been associated with an increase in oxidative stress in cells due to a larger glucose oxidation in the aerobic respiration pathways leading to an increased production of harmful ROS, which might negatively affect cell growth at high glucose concentrations. Meanwhile, cultures with 20 mM feed glucose concentration and at High-D had the highest viable cell densities, low q_GLC_, but the maximum Y_Lac/Glc_. While a highly proliferative phenotype is usually associated with a high q_GLC_ and q_LAC_ [61], it is interesting that the conditions with highest viable cell density presented low q_GLC_ and r-protein production when compared to other conditions, suggesting, thereby, that most of the consumed carbon which is not used for biomass is converted to lactate. Therefore, a low glucose concentration at high dilution rates causes a lower metabolic efficiency in this CHO cell line.

We also observed that the dilution rate plays a key role in modulating cell growth at low glucose concentrations, while at a high glucose concentration it is not particularly relevant. In fact, the dilution rate may constrain cell growth in cultures between 20 mM and 30 mM glucose, but at 40 mM glucose, there is no effect of dilution rate on cell growth. Hayer et al. observed that there are no significant changes in viable cell concentration when working at lower feed glucose concentrations (2.75 or 4.5 mM) and with dilution rates between 0.01 to 0.02 1/h [33]. This study also confirmed that there is practically no effect of temperature on cell growth when the dilution rate is manipulated, as reported by Vergara et al. [42]. This therefore suggests that the constrained cell growth under mild hypothermic conditions in batch culture [7,27] is mainly caused by the lower specific growth rate induced by low temperature.

Furthermore, cell cycle distribution was affected by the environmental conditions, showing an increased accumulation of cells in G1/G0, and decreasing proportions in S and G2/M, at the low dilution rate and mild hypothermia. Moreover, in all conditions, over 50% of cells remained at G1/G0. Otherwise, we observed cell accumulation in G2/M under 30 mM and 40 mM glucose concentration, compared to cultures with 20 mM glucose. To our knowledge, there is no previous report relating cell cycle arrest in G2/M due to changes in medium glucose concentration. A possible explanation for this phenomenon might lie in the cells’ capacity to sense their metabolic state and activate signaling pathways related to cell cycle. Kaplon et al. suggested that cell cycle progression is tightly regulated through crosstalk between signaling pathways and metabolism [62]. Furthermore, this explanation is supported by a recent study in mammalian cells reporting that fructose 2,6-bisphosphate (F2,6BP) acts as a glucose sensor regulating survival and cell cycle progression [62]. In fact, glucose deprivation reduces F2,6BP which, in turn, suppresses the activity of Cdks causing G1 arrest [63]. Therefore, in a situation that is the precise opposite, higher glycolysis activity due to an increase in glucose availability might increase F2,6BP causing cell cycle progression to G2/M. Meanwhile, decreasing both specific cell growth rate [64,65] and culture temperature [15,24,66,67] has been extensively demonstrated to arrest the cell cycle in G1.

As described previously, an increase of media glucose concentration coupled with a lower dilution rate led to an improvement in specific *rh*-tPA productivity. The increased q_*rh*-tPA_ observed in cultures at high glucose concentration was consistent with those reported in batch [11,36] and chemostat cultures [33,34,68]. The improvement of r-protein productivity as carbon source concentration increases has also been reported for other hexoses, such as mannose [34,47]. Recently, an upregulated expression of several proteins involved in the translation and folding processes and a downregulated expression of the proteins related to unfolded protein response have been observed in CHO cells cultured in high glucose medium[11]. These results therefore suggest that the improved specific *rh*-tPA productivity in high glucose media might be explained, in part, by the enhancement of translational machinery and the reduction of protein degradation in CHO cells.

Mammalian cell-based biopharmaceutical manufacturing is not recommended in high glucose containing media due to the potential glycosylation modifications on recombinant proteins [37] and the harmful effects of lactate overproduction on growth and productivity. However, mammalian cells used for biopharmaceutical production demand a large amount of glucose to simultaneously cope with growth and r-protein production [10]. That is why it has become paramount to determine an optimal glucose concentration in culture media that enables a compromise between growth and r-protein production.

A reduction in dilution rate also had a significant impact on r-protein productivity in this study. The increase in specific r-protein productivity at lower specific cell growth rates has been observed in CHO cells both in batch [21,47] and chemostat [42,69] cultures. A similar response was observed in chemostat cultures of hybridoma cells [44,70]. These observations are supported by two previous studies, indicating an inverse relationship between specific cell growth and r-protein production in two different CHO cell lines [6,47]. Tey and Al-Rubeai [71] suggested that at higher biomass synthesis rates due to higher specific cell growth rates, cell metabolism is not capable of supporting a higher r-protein production rate. However, at lower biomass synthesis rates there is a lower demand for energy and biomass building blocks, providing the opportunity to maximize r-protein production. This explanation is in line with our previous findings, showing that a reduction of specific cell growth can enhance specific *rh*-tPA productivity [42].

The improvement of specific r-protein productivity in cultures at low temperature is widely reported in batch culture [13–18], but not in CHO cell chemostat culture [42]. This discrepancy between the modalities lies in the fact that the increased specific r-protein productivity at low temperature is accompanied by a decline in the specific growth rate, which is not normally discussed. However, when the specific growth rate is fixed through the dilution rate in steady-state continuous cultures, there is no effect of low temperature on the specific r-protein productivity [42]. Our current results also enabled us to observe the temperature effect on specific r-protein productivity, as widely reported in batch systems, by comparing the culture at High-D and 37 °C (typical control culture in batch system) with the culture at Low-D and 33 °C (culture under mild hypothermia in batch system), both at 20 mM glucose (Fig 2). Nevertheless, when dilution rate was fixed, a negative impact of mild hypothermia on q_t-PA_ was observed at both High-D and Low-D, regardless of the glucose concentration. This evidence suggests that the increase in specific productivity of *rh*-tPA and probably other r-proteins under mild hypothermia is in part due to a cellular response to lower cell growth and more efficient glucose use. Hence, increasing glucose availability for cells and the assurance of a low biomass synthesis rate could be a strategy to enhance r-protein productivity in CHO cells, at least at the operational level.

A technical aspect to consider in the present study is that all cultures were performed in spinner flasks, which do not allow for monitoring and control of dissolved oxygen (DO) or pH. In previous studies based on mammalian cells, spinner flask-based cultures have proven to maintain dissolved oxygen tension (DOT) levels greater than 30% [67]. In addition, DOT levels between 30% and 90% air saturation in continuous cultures do not significantly affected growth or specific productivity of murine B-lymphocyte hybridoma cells [72]. Since our cultures achieved low viable cell densities (around 1 × 10^6^ cells/mL) at steady state, we presume that our cells do not present a high oxygen demand under the evaluated conditions. On the other hand, pH variation in mammalian cell cultures, which is mainly caused by the accumulation of lactate, has a negative impact on growth and specific productivity [25,73]. Our chemostat cultures showed a residual lactate concentration with values oscillating between 3 and 15mM (Table 3), which suggest the pH might decrease at different levels, depending on the steady state lactate concentration in each chemostat culture. However, concentrations below 20 mM lactate have been shown to not affect cell growth or productivity in CHO cells [54]. Although we cannot rule out the possibility that differences in DOT level or pH might affect culture performance, particularly when comparing single variations of an environmental variable, the low viable cell densities and reduced lactate accumulation suggests that DOT levels and pH change had a low impact on our cultures at steady state.

The consumption of carbon sources was substantially affected by environmental culture conditions. An increase of glucose concentration in the feed resulted in higher glucose and glutamate consumption rates in most cases. Similar responses in q_Glc_ due to high glucose media have been previously described in CHO cells [33,68], BHK cells [74], and a recombinant SP2/0 myeloma suspension cell line [75], thereby suggesting it is a common response across multiple mammalian cell lines. It has also been reported that an increase of glucose in the media did not cause a significant increase in activity of the key glycolytic enzymes (i.e. hexokinase, phosphofructokinase and pyruvate kinase) [32]. This brings to light the great capacity of glycolysis to metabolize glucose and the lack of control over the glycolytic enzyme that would be key to the high glucose tolerance of mammalian cell lines [68]. These observations also raise the question whether there are differential expression levels of glycolytic enzymes and glucose transporters in mammalian cell lines in media under high glucose concentration. Recently, Gowtham et al. [12] performed a transcriptome analysis in CHO cells at low (10 mM) and high (30 mM) glucose concentrations. Their results indicated that glucose concentrations between 10 and 30 mM do not have significant variation in the expression levels of genes related to central carbon metabolism.

Glutamate, as the replacement of glutamine, plays an important role in cell growth and cell survival. It is a major nitrogen source and also a main energy source in CHO cell cultures [76,77]. Our result of increased q_Glu_ with increase of glucose feed concentration was consistent with previous observations, where glutamate was used as a glutamine replacement [68]. However, glutamate consumption rates were between 10 and 20 times lower than glucose consumption rates in culture (Fig 3), thus suggesting that, in the range of studied culture conditions, glucose rather than glutamate generates the differences in cell growth and r-protein production in culture.

Unlike a high glucose concentration in medium, a low dilution rate did not significantly impact glucose consumption but impacted glutamate consumption, by increasing the q_Glu_ in cells. Similar responses were previously reported regarding unaltered glucose [42,71] and decreased glutamate [33,42] consumption against lower specific cell growth rates. However, when glucose in media was below 10 mM, different mammalian cell lines presented a reduction of q_Glc_ as glucose concentration in medium decreased [33,44,70,78]. On the other hand, mild hypothermia caused opposite responses in glucose and glutamate metabolisms, decreasing q_Glc_ except in 20 mM at High-D and increasing q_Glu_ also except in 20 mM at High-D. While reduced glucose metabolism under mild hypothermia has been widely described and explained in the literature [24,25,47,67,79], there has been no consensus on the response of glutamate metabolism to lower temperatures, having reported both an increase [19,42,67] and a decrease [25] of q_Glu_. A recent study evaluating the impact of mild hypothermia on the transcriptomic responses showed that *gls* and *asct2* encoding for glutaminase and glutamine transporter, respectively, were significantly overexpressed under mild hypothermia [8]. Such observations coincided with our results showing higher metabolism of glutamate at low temperature.

Specific lactate production was increased under high glucose availability in the feed, as expected. In chemostat cultures, higher glucose concentration in medium promoted lactate production in CHO cells [34,68] and hybridoma cells [75]. The same response was observed in batch cultures [10,11,29,32], where the increased q_Lac_ had been additionally accompanied by a 41% increase in lactate dehydrogenase (LDH) activity and limited cell growth [32]. On the contrary, reducing cell metabolism either by lowering feed glucose concentration from 40 to 20 and 30 mM or lowering culture temperature in most of cases (except at 20 mM) led to lower levels of q_Lac_ (see Fig 3). These cell responses were consistent with the reported data, both for lower specific cell growth rates [33,42,44,70,78] and culture temperatures [24,25,67]. Although previous studies on transcriptome analysis in CHO cells under mild hypothermia reported increased expression of mRNA encoding two LDH isoforms [8,80,81], this does not necessarily lead to an increased LDH activity or q_Lac_.

Through multivariate analysis, we elucidated the considerable effect that glucose has on the variability of CHO cell culture performance. On the one hand, glucose consumption rate was the pivotal physiological parameter affecting volumetric productivity, since it had an important impact on specific *rh*-tPA productivity and cell growth (Fig 4A). On the other hand, glucose availability was the most influential culture condition, enabling the differentiation of clusters (Fig 4B). Several studies have indicated the influential role glucose plays in the expression of proteins involved in metabolism, cell growth and enzyme activity [10,11,32]. However, none of these studies have associated the differential impact of glucose with culture performance using a multivariate analysis. Therefore, glucose is certainly a relevant player in designing a cell culture bioprocess.

PCA revealed that increases in specific r-protein productivity were not related to an increase in the number of cells in G1/G0 phase. The increase of heterologous protein expression via the manipulation of cell cycle, particularly with G1 arrest, of recombinant mammalian cells is one of the most described strategies [13,15,19,24,67,82]. Despite the well-documented relationship between cell cycle arrest in G1 and increased r-protein productivity, the reasons underlying such increases remains unclear. Cells arrested at the end of the G1-phase of cell cycle present a more active metabolism, are larger in size [83,84], and have more active transcriptional machinery [85] than non-arrested cells. However, all previous studies have been performed in batch culture by manipulating either environmental culture conditions (i.e. temperature shift or chemicals supplementation in medium) or cell-engineering based approaches for cell cycle arrest [82]. So, it becomes practically impossible to distinguish between the different approaches and their effects on cell growth. In the present study, we have successfully isolated the effect of specific cell growth, culture temperature and media glucose concentration on r-protein production and provided evidence that the real modulator of r-protein production is the specific cell growth rate regardless of the distribution of the cell population in each specific cell cycle phase.

## Conclusion

The principal aim of this study was to investigate the individual and combined impacts of feed glucose concentration, dilution rate and temperature on culture performance and cell metabolism of a *rh*-tPA producing CHO cell line using chemostats. Our results indicated significant changes in cell growth, cycle distribution, metabolism, and *rh*-tPA productivity due to the manipulation of operational variables. Increasing glucose concentration in media led to a constrained cell growth, increased specific *rh*-tPA productivity, and arrest in the G2/M cell cycle phase. Meanwhile, lowering dilution rate or lowering culture temperature reduced the metabolic parameters, namely glucose consumption and lactate production rates. Considering all evaluated conditions, our findings suggest that a reduced dilution rate coupled with a high glucose concentration in medium significantly improves r-protein productivity in CHO cells. We also confirmed that mild hypothermia had no effect on specific *rh*-tPA productivity, regardless of the glucose concentration or dilution rate. Multivariate analysis on one side revealed that for glucose, both its concentration in feeding media and consumption by cells had the highest impact on kinetic parameter variability. This allowed us to question the true relationship between specific *rh*-tPA productivity, hypothermia, and arrest of cells in G1/G0 phase observed in batch culture. On the other hand, our results indicated that higher productivity is related to a lower dilution rate, while mild hypothermia is related to the arrest of cells in the G1/G0 phase, without a direct impact on the increase in specific productivity observed by others. The present study successfully elucidated the individual impacts of three operational variables, supporting the fact that specific growth rate as a function of carbon source concentration is the most important variable during the development of a bioprocess for increased productivity, at the macroscopic level. It should be mentioned that this might be clone and protein product dependent. Therefore, our future research aims to understand the cellular responses associated with increased productivity from a transcriptomic and proteomic perspective. We also aim to analyze other recombinant proteins produced in different cell lines.
